# Necessary and sufficient conditions for exact closures of epidemic equations on configuration model networks

**DOI:** 10.1007/s00285-023-01967-9

**Published:** 2023-08-02

**Authors:** István Z. Kiss, Eben Kenah, Grzegorz A. Rempała

**Affiliations:** 1 Department of Mathematics, University of Sussex, Falmer, Brighton, BN1 9QH UK; 2 Network Science Institute, Northeastern University London, London, E1W 1LP UK; 3 Division of Biostatistics, College of Public Health and Mathematical Biosciences Institute, The Ohio State University, Columbus, OH USA

**Keywords:** Epidemics, Networks, Inference, Pairwise models, Survival analysis, 00A71, 37N25, 92D25

## Abstract

We prove that it is possible to obtain the exact closure of SIR pairwise epidemic equations on a configuration model network if and only if the degree distribution follows a Poisson, binomial, or negative binomial distribution. The proof relies on establishing the equivalence, for these specific degree distributions, between the closed pairwise model and a dynamical survival analysis (DSA) model that was previously shown to be exact. Specifically, we demonstrate that the DSA model is equivalent to the well-known edge-based Volz model. Using this result, we also provide reductions of the closed pairwise and Volz models to a single equation that involves only susceptibles. This equation has a useful statistical interpretation in terms of times to infection. We provide some numerical examples to illustrate our results.

## Introduction

Many models of the transmission dynamics of infectious diseases (e.g., Khuda Bukhsh et al. 2022; Kiss et al. 2017; Gross et al. 2006; Ball et al. 2019; Risau-Gusmán and Zanette 2009; Jacobsen et al. 2018) represent contacts as a random graph of *N* individuals (nodes) formed using the configuration model (Molloy and Reed 1995; Bollobás 2001). Node degrees are typically assumed to be independent and identically distributed, as in the Newman–Strogatz–Watts (NSW) random graph construction Newman et al. (2001). Unfortunately, the systems of equations required to fully describe the stochastic dynamics of epidemics on such networks are often too large to handle, even for moderate values of *N*. A common simplification is to average stochastic quantities, but this still often leads to infinite-sized systems of equations. To address this challenge, some authors have employed a “closure” technique to create a reduced and closed (finite) system of equations by expressing terms corresponding to larger structures in terms of smaller structures. In most cases, this representation involves an approximation, but it can be exact in the case of SIR dynamics on configuration model networks, as demonstrated in Kiss et al. (2017).

Approximating stochastic epidemics on networks is an important problem that has received significant attention, leading to the development of several mean-field models that are represented in terms of systems of ordinary differential equations. The pairwise model Rand (1999a); Keeling (1999) is based on a set of equations for the expected number of susceptible ([*S*]) and infected ([*I*]) nodes and the expected number of S–I ([*SI*]) and S–S ([*SS*]) pairs. It relies on a closure that approximates expected number of triples in terms of singles and pairs, which breaks the dependence on ever-higher moments. The Volz model Volz (2008) is based on a system of differential equations that relies on the probability generating function (PGF) of the degree distribution as well as edge-centric quantities (such as the number of edges with nodes in certain states) rather than node-centric quantities (such as the number of infected or susceptible nodes). This model gave excellent agreement with simulations, and it was formally proven to be the large-*N* limit of a stochastic SIR epidemic on a configuration model network by Decreusefond and colleagues Decreusefond et al. (2012) More recently, Jacobsen and colleagues Jacobsen et al. (2018) provided an alternative method to derive the mean-field limit of a stochastic SIR model on a multi-layer network that we refer to as dynamical survival analysis (DSA). This approach results in a mean-field model over variables different from those in Volz’s approach, but it also shows the exactness of the Volz model in the large-network limit. The DSA formulation allows us to reinterpret the epidemic from a statistical viewpoint (e.g., by approximating the probability that a typical node who was susceptible at time $t = 0$ is still susceptible at time $t > 0$).

In this paper, we show that the pairwise model closure is exact if and only if the contact network has a degree distribution that is Poisson, binomial, or negative binomial (which we call *Poisson-type* distributions). Once this condition is satisfied, the limiting pairwise model closure is equivalent to the edge-based model proposed by Volz (2008) and extended by Miller (2011) as well as to the network-based DSA model KhudaBukhsh et al. (2020). We also show that the equivalence between the Volz–Miller and DSA models holds for any degree distribution with finite variance and that it allows statistical inference for epidemic model, as applied recently to early COVID-19 pandemic modelling Di Lauro et al. (2022); KhudaBukhsh et al. (2023).

The rest of the paper is organised as follows: In Sect. 2, we briefly describe stochastic epidemic dynamics on a configuration model network along with limiting approximations based on the pairwise, Volz, and DSA approaches. In Sect. 3, we introduce and characterise the class of Poisson-type distributions and then present our main result on the necessary and sufficient condition for the exact closure of the pairwise network model. This result is more precise than that obtained in Jacobsen et al. (2018), but it is less general as it only covers single-layer networks. In Sects. 4 and 5, we provide additional details on DSA model’s connection with statistical inference and offer concluding remarks. Additional calculations on the DSA and Volz models are presented in the Appendix.

## Network epidemic models

We describe the underlying dynamics of the stochastic SIR epidemic process on a network of size *N* as follows: At the start of an epidemic, we pick *m* initially infectious individuals at random from the population. An infectious individual remains so for an infectious period that is sampled from an exponential distribution with rate $\gamma$. During this period, s/he makes contact with his or her immediate neighbours according to a Poisson process with intensity $\beta$. If a contacted individual is still susceptible at the time of the contact, s/he will immediately become infectious. After the infectious period, the infectious individual recovers and is immune to further infections. All infectious periods and Poisson processes are assumed to be independent of each other.

The epidemic is assumed to evolve on a configuration model network that is constructed as follows: Each node is given a random number of half edges according to a specified degree distribution $(p_k)$, and all half edges are matched uniformly at random to form proper edges.^1^ Although the exact behaviour of this SIR epidemic process is quite complicated, there exist several approximations that rely on aggregated or averaged quantities. To describe them, generating functions are useful.

### Probability generating function

If $p_k$ is the probability that a randomly chosen node has degree *k*, then the probability generating function (PGF) of the degree distribution is

$$\begin{aligned} \psi (u) = \sum _{k = 0}^\infty p_k u^k. \end{aligned}$$

The PGF $\psi$ contains a tremendous amount of information about epidemic dynamics on configuration model networks. Let $\theta$ be the probability that an initially susceptible node of degree one remains uninfected at time *t* in an infinite network. Then, assuming no variation in infectiousness or susceptibility to infection among nodes except for their degree, the probability that a node with degree *k* remains uninfected equals the probability $\theta ^k$ that infection has not crossed any of its edges (see Volz (2008)). Summing over all possible *k* shows that $\psi (\theta )$ is the probability that a randomly chosen node remains susceptible in an infinite network. The degree distribution of the remaining susceptible nodes has the PGF

1 $$\begin{aligned} u \mapsto \frac{\sum _{k = 0}^\infty (p_k \theta ^k) u^k}{\sum _{k = 0}^\infty p_k \theta ^k} = \frac{\psi (\theta u)}{\psi (\theta )}, \end{aligned}$$

which equals $\psi (u)$ when $\theta = 1$. Via (1), $\psi$ tells us about the properties of a node chosen uniformly at random from the set of susceptible nodes.

The first derivative of $\psi$ tells us about the mean degree of susceptible nodes and about the properties of a node reached by crossing an edge. At a given value of $\theta$, the mean degree of the remaining susceptible nodes is

2 $$\begin{aligned} \frac{\text {d}}{\text {d} u} \frac{\psi (\theta u)}{\psi (\theta )} \bigg |_{u = 1} = \frac{\theta \psi '(\theta )}{\psi (\theta )}, \end{aligned}$$

which equals $\psi '(1)$ when $\theta = 1$. If we cross an edge, the probability that we end up at a node with degree *k* is proportional to *k*. If we start at a node chosen uniformly at random and cross an edge to reach a neighbour, the number of edges we can use to reach a third node has the PGF

$$\begin{aligned} u \mapsto \frac{\sum _{k = 1}^\infty (k p_k) u^{k - 1}}{\sum _{k = 1}^\infty k p_k} = \frac{\psi '(u)}{\psi '(1)}. \end{aligned}$$

If you are a susceptible node with a neighbour of degree *k*, this neighbour remains susceptible as long as infection has not crossed any of the $k - 1$ edges that lead to a third node. Thus, the probability that a neighbour of a susceptible node remains susceptible is $\psi '(\theta ) / \psi '(1)$. If we cross an edge to reach a susceptible neighbour, the number of edges we can cross to reach a third node has the PGF

$$\begin{aligned} u \mapsto \frac{\sum _{k = 1}^\infty \big (k p_k \theta ^k\big ) u^{k - 1}}{\sum _{k = 1}^\infty k p_k \theta ^k} = \frac{\psi '(\theta u)}{\psi '(\theta )}. \end{aligned}$$

This distribution is often called the *excess degree distribution* (at the time *t* corresponding to $\theta$), and it plays an important role in the dynamics of epidemics on networks. At a given value of $\theta$, the mean excess degree of susceptible nodes is

3 $$\begin{aligned} \frac{\text {d}}{\text {d} u} \frac{\psi '(\theta u)}{\psi '(\theta )} \bigg |_{u = 1} = \frac{\theta \psi ''(\theta )}{\psi '(\theta )}, \end{aligned}$$

where $\psi ''$ represents the second derivative of $\psi$.

### Pairwise model

The pairwise model provides an intuitive way of describing the dynamics of an SIR epidemic on a configuration model graph. The pairwise model equations, as proposed for instance in Rand (1999a), are:

4 $$\begin{aligned} \begin{aligned} {[}\dot{S}]&= -\beta [SI], \\ {[}\dot{I}]&= \beta [SI]-\gamma [I], \\ {[}\dot{R}]&= \gamma [I], \\ {[}\dot{SI}]&= -\gamma [SI] + \beta \big ([SSI] - [ISI]\big ) - \beta [SI], \\ {[}\dot{SS}]&= -2 \beta [SSI], \end{aligned} \end{aligned}$$

where [*A*], [*AB*], [*ABC*] with $A, B, C \in \{S, I, R\}$ stand for the number of singles, doubles and triples in the entire network with the given sequence of states when each group is counted in all possible ways. More formally,

5 $$\begin{aligned}{}[ABC] = \sum _{i = 1}^N \sum _{j = 1}^{N} \sum _{k=1}^N a_{ij} a_{jk} I_i(A) I_j(B) I_k(C), \end{aligned}$$

where $(a_{ij})_{i,j=1,2,\dots , N}$ is the adjacency matrix of the network with entries either zero or one and $I_i(A)$, $I_i(B)$, and $I_i(C)$ are binary variables that equal one when the status of *i*-th individual is *A*, *B*, and *C*, respectively, and equal zero otherwise. The singles [*A*] and doubles [*AB*] are similarly defined. We consider undirected networks with no self-loops, so $a_{ii}=0$ and $a_{ij}=a_{ji}$.

To completely describe the model, additional equations for triples are needed. These will depend on quadruples, which will depend on quintuples, and so on. To make the model tractable in the face of an ever-increasing number of variables and equations, one often introduces the notion of a “closure” in which larger structures (e.g., triples) are represented by smaller ones (e.g. pairs). The model (4) can be closed using the methods described in Sect. 3.

The two models that we describe next do not require closure and are known to be exact in the large network limit (i.e., as $N \rightarrow \infty$) Decreusefond et al. (2012); Bohman and Picollelli (2012); Barbour and Reinert (2013); Janson et al. (2014). However, they are less straightforward to interpret.

### Volz’s model

In addition to the limiting ($N\rightarrow \infty$) probability $\theta$ defined in Sect. 2.1, let us also introduce the limiting probabilities $p_I$ and $p_S$ that a randomly selected edge with one susceptible vertex is of type *SI* and *SS*, respectively. In this notation, Volz’s mean-field equations Volz (2008) are:

6 $$\begin{aligned} \dot{\theta }= & {} -\beta p_{I} \theta , \nonumber \\ \dot{p}_{I}= & {} \beta p_{S} p_{I} \theta \frac{\psi ''(\theta )}{\psi '(\theta )} - \beta p_{I} \left( 1 - p_{I}\right) - \gamma p_{I}, \nonumber \\ \dot{p}_{S}= & {} \beta p_{S} p_{I} \left( 1 - \theta \frac{\psi ''(\theta )}{\psi '(\theta )}\right) , \nonumber \\ x_S= & {} \psi (\theta ), \nonumber \\ \dot{x}_I= & {} \beta p_{I} \theta \psi '(\theta ) - \gamma x_I, \end{aligned}$$

where the derivative with respect to time is marked with a dot and the derivative with respect to $\theta$ is marked with a prime. Here $x_S$ and $x_I$ denote the limiting ($N\rightarrow \infty$) proportions of susceptibles and infected, respectively. Note that the first three equations are decoupled from the remaining two and that the proportion of recovered may be obtained from the conservation relationship. The initial conditions are

7 $$\begin{aligned} \begin{aligned} x_S(0) = \theta (0) = p_S(0)&= 1,\\ x_I(0) = p_I(0)&= \rho , \end{aligned} \end{aligned}$$

where $$0 < \rho \ll 1$$.

### DSA model

An alternative description of the limiting dynamics of a large configuration model network under an SIR epidemic was given in Jacobsen et al. (2018). Although originally considered in the context of multi-layer networks, its single layer version has been applied recently to statistical inference problems under the name “dynamical survival analysis” (DSA) KhudaBukhsh et al. (2023). In this approach, the limiting equations are derived in terms of the limiting ($N\rightarrow \infty$) proportions $x_{SI}$ of *SI*-type and $x_{SS}$ of *SS*-type edges and the additional quantity $x_\theta$. In Appendix B, we show that the latter coincides with the probability $\theta$ defined in Sect. 2.1. The equations are:

8 $$\begin{aligned} \begin{aligned} \dot{x}_\theta&= -\beta \frac{x_{SI}}{ \psi '\left( x_\theta \right) }, \\ \dot{x}_{SS}&= -2 \beta x_{S I} x_{S S} \frac{ \psi ''\left( x_\theta \right) }{ \psi '\left( x_\theta \right) ^{2}}, \\ \dot{x}_{SI}&= x_{S I}\Bigg [\beta \big (x_{S S}-x_{S I}\big ) \frac{ \psi ''\left( x_\theta \right) }{ \psi '\left( x_\theta \right) ^{2}} - (\beta + \gamma )\Bigg ], \\ \dot{x}_{S}&= -\beta x_{S I}, \\ \dot{x}_{I}&= \beta x_{S I}-\gamma x_{I}. \end{aligned} \end{aligned}$$

As in Volz’s system, the first three equations do not depend explicitly on the dynamics of $x_S$ and $x_I$ and therefore may be decoupled from the remaining two equations. The initial conditions are:

9 $$\begin{aligned} \begin{aligned} x_{S}(0) = x_{\theta }(0)&= 1, \\ x_{I}(0)&= \rho , \\ x_{SS}(0)&= \mu , \\ x_{SI}(0)&= \mu \rho , \end{aligned} \end{aligned}$$

where $$0 < \rho \ll 1$$ and $$\mu > 0$$.

## Closing the pairwise model

In practice, one needs to define the time dynamics of the triples [*SSI*] and [*ISI*] to use the system (4). Typically, these equations are closed by approximating the dynamics of triples using pairs. This method is referred to as the “triples closure” in House and Keeling (2011) and the “pair approximation” or “pairwise closure” in Jacobsen et al. (2018).

### Exact closure condition

While various justifications of closures have been proposed before Kiss et al. (2009), we present a slightly different justification that is focused on the PGF of the degree distribution. The form of the degree distribution plays a key role in obtaining necessary and sufficient conditions on the network to ensure that pairwise closures are exact.

Let $[A_j B_k C_\ell ]$ indicate the number of connected triplets *ABC* as defined in Eq. (5) such that the node in state *A* has degree *j*, the node in state *B* has degree *k*, and the node in state *C* has degree $\ell$. Then

10 $$\begin{aligned} {[}ABC] = \sum _{j,k,l} [A_j B_k C_\ell ] \end{aligned}$$

and similarly for [*A*] and [*AB*]. Let us also define $[S \bullet ]:= [SS]+[SI]$. We derive a closure condition starting from the finest resolution, where we account for the degree of each node. Let state *A* below denote either *S* or *I*. We are interested in approximating [*ASI*]. Note that we may approximate, in two stages, as follows:

11 $$\begin{aligned}{}[A_j S_k I_\ell ] \simeq (k - 1) [A_j S_k] \frac{[S_k I_\ell ]}{k [S_k]} \simeq (k - 1) [A_j S_k] \frac{[S I_\ell ]}{[S \bullet ]}, \end{aligned}$$

where we assume that a degree *k* susceptible node’s neighbour is as likely to be infected as any other susceptible node’s neighbour. Intuitively, the first approximation is valid because we start with an $A_j S_k$ pair and each of the $k - 1$ additional edges connected to the $S_k$ node leads to an $I_\ell$ node with probability $[S_k I_\ell ] / (k [S_k])$. The second approximation follows from the configuration model. These will be used repeatedly in what follows. Summing over the $\ell$ index alone we get:

12 $$\begin{aligned}{}[A_jS_kI] = \sum _{\ell } [A_j S_k I_\ell ] \simeq \sum _{\ell } (k - 1) [A_j S_k] \frac{[S I_\ell ]}{[S \bullet ]} \simeq (k - 1) [A_j S_k] \frac{[S I]}{[S \bullet ]}. \end{aligned}$$

A similar approximation to $[A_j S_k]$ and summation over *j* leads to $[AS_k] \simeq [AS]\times (k[S_k]/[S\bullet ])$. This, in turn, leads to:

13 $$\begin{aligned}{}[A S_k I] = \sum _{j} [A_j S_k I]= & {} \sum _{j} (k - 1) [A_j S_k] \frac{[S I]}{[S \bullet ]} \simeq (k - 1) [A S_k] \frac{[S I]}{[S \bullet ]}\nonumber \\\simeq & {} (k - 1) k[S_k] \frac{[A S][S I]}{[S \bullet ]^2}. \end{aligned}$$

Finally, summing over *k* leads to:

14 $$\begin{aligned} {[}ASI]=\sum _{k}[AS_kI]= \sum _{k}(k-1)k[S_k]\frac{[AS][SI]}{[S\bullet ]^2}\simeq \frac{[AS][SI]}{[S\bullet ]^2}\sum _{k}(k-1)k[S_k].\nonumber \\ \end{aligned}$$

It remains to handle $\sum _k k (k - 1) [S_k]$.

Recall that the variable $\theta$ ($x_\theta$ in the DSA model) is the probability that infection has not crossed a randomly chosen edge, and it decreases over time. At a given value of $\theta$, a node of degree *k* remains susceptible with probability $\theta ^k$, so $[S_k] \simeq N p_k \theta ^k$ where $p_k$ is the probability mass on *k* in the degree distribution. In terms of the degree distribution PGF $\psi$, for large *N* we have approximately (see, for instance, House and Keeling (2011); Jacobsen et al. (2018)):

15 $$\begin{aligned} \begin{aligned} {[}S]&\simeq N \psi (\theta ), \\ [S \bullet ] \simeq \sum _k k N p_k \theta ^k&= N \theta \psi '(\theta ), \\ \sum _{k} (k - 1) k [S_k]&\simeq N \theta ^2 \psi ''(\theta ). \end{aligned} \end{aligned}$$

Using these and (14) leads to:

16 $$\begin{aligned}{}[ASI]\simeq \frac{\psi ''(\theta )\psi (\theta )}{\psi '(\theta )^2}\frac{[AS][SI]}{[S]}. \end{aligned}$$

Because $\theta$ is a new dynamic variable, an equation for it is needed. However, we need no more equations as long as

17 $$\begin{aligned} \frac{\psi ''(\theta ) \psi (\theta )}{\psi '(\theta )^2} = \kappa = \text { constant}, \end{aligned}$$

in which case the dependency on $\theta$ is curtailed. If $\theta$ is the probability that disease has not crossed a randomly chosen edge, then it follows from Eqs. (2) and (3) that the left-hand side of Eq. (17) is the mean excess degree of susceptible nodes divided by their mean degree. Therefore, the condition above simply implies that this ratio remains constant as the susceptible nodes are depleted over time. Below, we show that the networks for which this property holds can be explicitly characterized. Remarkably, for such networks, (17) is equivalent to the exact closure—that is to the asymptotic ($N \rightarrow \infty$) equality in (16).

### Poisson-type distributions

Assuming that $\psi (0),\psi ^\prime (0) >0$, it follows that $\psi ,\psi ^\prime >0$ on the domain [0, 1] and the condition (17) can be rewritten as

18 $$\begin{aligned} \frac{\psi ''(u)}{\psi '(u)} =\kappa \frac{\psi '(u)}{\psi (u)}. \end{aligned}$$

for any $u \in [0,1]$. Upon integrating, we get the first-order differential equation

19 $$\begin{aligned} \psi ^{\prime }(u) = \alpha \, \psi (u)^\kappa \end{aligned}$$

for arbitrary constants $\alpha > 0$ and $\kappa > 0$. Because $\psi$ is analytic, the equation above is defined beyond the original domain—in particular in the small right-side neighbourhood of the natural initial condition $\psi (1) = 1$.

Table 1 below presents a family consisting of three distributions whose PGFs satisfy the ODE (19). We refer to these distributions as *Poisson-type* (PT) distributions. It turns out that being the PGF of a PT distribution is necessary and sufficient for (19) to hold:

#### Theorem 1

(Characterization of the Poisson-type distributions). The PGF of a random variable satisfies (19) if and only if the random variable belongs to the PT family listed in Table 1.

#### Proof

If $\kappa = 1$, the ODE given by (19) and the PGF condition $\psi (1) = 1$ imply that $\psi (u) = e^{u (\alpha - 1)}$, which is the PGF of the Poisson random variable $\textrm{POI}(\alpha )$ in Table 1. If $\kappa \ne 1$, separating variables and integrating gives us

20 $$\begin{aligned} \frac{\psi (u)^{1 - \kappa }}{1 - \kappa } = \alpha u+c, \end{aligned}$$

for some constant *c*. Taking into account the condition $\psi (1) = 1$, we get

21 $$\begin{aligned} \psi (u) = [\alpha (1 - \kappa ) (u - 1) + 1]^{\frac{1}{1 - \kappa }}. \end{aligned}$$

Now, consider separately the cases $\kappa < 1$ and $\kappa > 1$.

- *Case* $\kappa \in (0, 1)$ Because $\psi ^{(s)}(0) \ge 0$ for each integer $s \ge 0$, we must have $n = (1 - \kappa )^{-1}$ is a positive integer and $\alpha (1 - \kappa ) \le 1$. Writing
  22 $$\begin{aligned} \psi (u) = \big [1 - \alpha (1 - \kappa ) + \alpha (1 - \kappa ) u\big ]^n, \end{aligned}$$
  we recognise $\psi (u)$ as the PGF of the binomial random variable $\textrm{BINOM}(n,p)$ with $p=\alpha (1 - \kappa )$. Note that we allow here for a degenerate distribution with $p=1$, which corresponds to $\psi (u)=u^n$, that is, an *n*-regular degree distribution.
- *Case* $$\kappa > 1$$ Writing
  23 $$\begin{aligned} \psi (u) = \left[ \frac{\frac{1}{\alpha (\kappa - 1) + 1}}{1 - \frac{\alpha (k - 1)}{\alpha (\kappa - 1) + 1}u}\right] ^{\frac{1}{\kappa -1}}, \end{aligned}$$
  we recognise $\psi (u)$ as the PGF of the negative binomial distribution $\textrm{NB}(r,p)$ with $r=\frac{1}{\kappa - 1}$ and $p=\frac{\alpha (\kappa -1)}{\alpha (\kappa - 1) + 1}$. Note that here necessarily $p<1$.

Thus by considering all possible values of $\kappa$, it follows that there are only three PGF solutions to Eq. (19) corresponding to the families of random variables listed in Table 1. $\square$

Note that although the distributions in Table 1 describe all possible solutions to (19), the equivalence of (17), (18), and (19) holds only under the conditions $\psi (0)>0, \psi ^\prime (0)>0$. This excludes the special case $p = 1$ in the family $\textrm{BINOM}(n, p)$ corresponding to an *n*-regular degree distribution. However, for that particular case, we have $\psi (u) = u^n$, which also satisfies (17) (but not 18).

**Table 1:** The PT random variables whose PGFs satisfy (19)

Condition	Family	Parameters
$\kappa \in (0, 1)$	Binomial: $\textrm{BINOM}(n,p)$	$n=\frac{1}{1-\kappa }$, $p=\alpha (1-\kappa )$
$\kappa = 1$	Poisson: $\textrm{POI}(\lambda )$	$\lambda =\alpha$
$\kappa >1$	Negative binomial: $\textrm{NB}(r,p)$	$r=\frac{1}{\kappa -1}$, $p=\frac{\alpha (\kappa -1)}{\alpha (\kappa -1)+1}$

### Closure theorem and models equivalence

We are now in a position to state the main result on the exactness of the pairwise closure. Here, we use “exactness” in the sense defined in Jacobsen et al. (2018), or, equivalently, in Janson et al. (2014). In both cases, the notion implies that the appropriately scaled stochastic vector of susceptibles, infecteds, and recovereds tends in an appropriate sense to a deterministic vector whose components are described by the system of ordinary differential equations given by (6) or (8). Yet another equivalent definition of the exact closure is that the equality in the triple approximation condition (24) holds upon dividing both its sides by *N* and taking the limit $N \rightarrow \infty$.

#### Theorem 2

(Exact pairwise closure). Assume that either $\psi (0)>0, \psi ^\prime (0)>0$, and $\psi ^{\prime \prime }(1)<\infty$, or that $\psi (u)=u^n$. The closure condition

24 $$\begin{aligned} {[}ASI]\simeq \kappa \frac{[AS][SI]}{[S]} \end{aligned}$$

for $A \in \{S, I\}$ in the pairwise model given by system (4) is exact (that is, equality in (24) with both sides multiplied by $N^{-1}$ holds asymptotically as $N\rightarrow \infty$) if and only if the underlying configuration model network has a Poisson-type (PT) degree distribution. Furthermore, $\kappa = (n - 1) / n < 1$, $\kappa = 1$, or $\kappa = (r + 1) / r > 1$ if the degree distribution is $\textrm{BINOM}(n,p)$, $\textrm{POI}(\lambda )$, or $\textrm{NB}(r,p)$, respectively.

#### Proof

Consider first evaluation of the term $\psi ''\left( x_{\theta }\right) / \psi '\left( x_{\theta }\right) ^{2}$. In Table 2, we show that this term is equivalent to $\kappa /x_{S}$ for all PT distributions. With this in mind, we are ready to show the equivalence between the limiting pairwise model and the DSA models under (24). In view of Theorem 1, this suffices to establish the current result.

**Table 2:** Resolving the $\psi ''\left( x_{\theta }\right) / \psi '\left( x_{\theta }\right) ^{2}$ term in the DSA equations for binomial, Poisson, and negative binomial distributions

	Binomial	Poisson	Negative binomial
Parameter(s)	(*n*, *p*)	$\lambda$	(*r*, *p*)
$\psi (x)$	$(1 - p + p x)^n$	$e^{\lambda (x - 1)}$	$\big (\frac{1-p}{1-p x}\big )^{r}$
$\psi '(x)$	$n p (1 - p + p x)^{n - 1}$	$\lambda e^{\lambda (x - 1)}$	$\frac{r p (1 - p)^r}{(1 - p x)^{r + 1}}$
$\psi ''(x)$	$n (n - 1) p^2 (1 - p + p x)^{n-2}$	$\lambda ^2 e^{\lambda (x - 1)}$	$\frac{r (r + 1) p^2 (1 - p)^r}{(1 - p x)^{r + 2}}$
$\frac{\psi ''(x_{\theta })}{\psi '(x_{\theta })^2}$	$\frac{n-1}{n} \times \frac{1}{x_S}$	$1 \times \frac{1}{x_S}$	$\frac{r + 1}{r} \times \frac{1}{x_S}$
$\kappa$	$\frac{n - 1}{n}$	1	$\frac{r + 1}{r}$

Note that $$\psi (x_{\theta })=x_S$$

Let us show equivalence between the evolution equations for [*SI*] and $x_{SI}$. Recall that $x_{A} =\lim _{N\rightarrow \infty } [A]/N$ and $x_{AB} =\lim _{N\rightarrow \infty } [AB]/N$ and that these limits exist uniformly in probability over any finite time interval Jacobsen et al. (2018). From the equation for *SI* in the pairwise model and (24)

25 $$\begin{aligned} \begin{aligned} \dot{[SI]}&= \beta \big ([SSI] - [ISI]\big ) - [SI](\beta + \gamma ) \\&= [SI] \left[ \beta \big ([SS] - [SI]\big ) \frac{\kappa }{[S]} - (\beta + \gamma )\right] . \end{aligned} \end{aligned}$$

Dividing both sides of the last equation by *N*, taking the limit $N \rightarrow \infty$ [which can be done in view of the appropriate LLN, see Jacobsen et al. (2018)], and using the fact that $\psi ''\left( x_{\theta }\right) / \psi '\left( x_{\theta }\right) ^2 = \kappa / x_S$, we arrive at:

26 $$\begin{aligned} \begin{aligned} \dot{x}_{SI}&= x_{SI} \bigg [\beta \left( x_{S S}-x_{S I}\right) \frac{\kappa }{x_{S}}-(\beta +\gamma )\bigg ] \\&= x_{SI}\left[ \beta \left( x_{S S} - x_{S I}\right) \frac{\psi ''\left( x_{\theta }\right) }{\psi '\left( x_{\theta }\right) ^2} - (\beta + \gamma )\right] \end{aligned} \end{aligned}$$

which is identical to the equation for $x_{SI}$ in the DSA model. We note that, when the degree distribution is PT, the equation for $x_{\theta }$ is no longer needed and the equivalences of the remaining equations follow similarly as above. For instance, from (24) and $\dot{[SS]} = -2 \beta [SSI]$ it follows that $\dot{x}_{SS} = -2 \beta \kappa x_{SS} x_{SI} /x_S = -2 \beta x_{SS} x_{SI}\psi ''\left( x_{\theta }\right) /\psi '\left( x_{\theta }\right) ^2$. The exactness of the DSA model Jacobsen et al. (2018) as the limit of the stochastic SIR model on a configuration network, implies thus the exactness of the scaled pairwise model (and (24)) as $N\rightarrow \infty$. $\square$

**Fig. 1:**
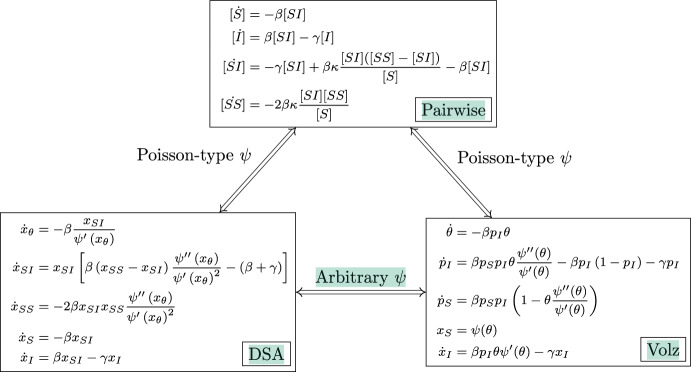
Summary of model equivalence results. The top is the pairwise model, the bottom left is the DSA model, and the bottom right is the Volz model

Figure 1 summarises the equivalences between models. The equivalence of the Volz model and the DSA model for an arbitrary degree distribution is shown in the Appendix.

**Fig. 2:**
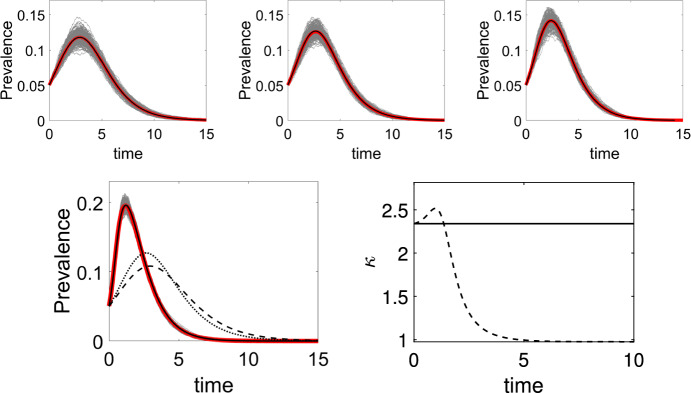
Top row: From left to right, epidemics on networks with regular (each node has $n = 6$ links), Poisson ($\lambda = 10$) and negative binomial ($r = 10$ and $p = 1/2$) degree distributions are plotted, respectively. Individual stochastic realisations are plotted with thin grey lines, their mean with the thick red line, and the solution of the corresponding pairwise model with a solid black line. Bottom left: Epidemics on a network where 80% of nodes have degree 4 and the rest have degree 34. The DSA model (solid black line) is used to match the average epidemics. The pairwise model closures with $\kappa = (n - 1) / n$ (dashed line) and $\kappa = 1$ (dotted line) are also plotted. Bottom right: Plot showing that, for a non-PT network (like the network with two distinct value for the degree of nodes), $\kappa$ is not constant in time. The value of $\kappa$ at time $t=0$ (solid constant line) is short lived as shown $\kappa (t)$ (dashed line) as given in Eq. (17). Other parameters are: $N = 10{,}000$ nodes, recovery rate $\gamma = 1$, and per-contact transmission rate $\beta = 0.4$ for the regular network and $\beta = 0.2$ for the networks with Poisson, negative binomial, and mixed degree distributions. Epidemics start with 250 infected nodes chosen at random and only epidemics reaching 500 infected individuals are retained. We average over 15 network realisations and 15 epidemics on each network (colour figure online)

The top row of Fig. 2 shows numerical evidence of the exactness of the closure in the pairwise model for PT networks. For PT networks, the agreement between the pairwise model and the expected value of explicit stochastic simulations is excellent. The DSA model continues to work well for non-PT networks (see bottom row of Fig. 2), and it is clear that $\kappa$ is not constant in time in this case. As expected, this means that none of the three possible closures work. In the left panel of the bottom row we plot the output from the pairwise model for $\kappa = (n - 1) / n$ (dashed line) and $\kappa = 1$ (dotted line). Both underestimate prevalence which in this case is driven by the 20% of highly connected nodes. This is captured poorly by both closures.

## Survival analysis perspective

The exact closure condition implies that, under the assumption of a PT degree distribution, the pairwise and DSA models are equivalent. One of the benefits of this equivalence is that the pairwise model shares the statistical interpretation of the DSA model. Indeed, as shown in KhudaBukhsh et al. (2020) [see also discussion with examples in Choi et al. (2019); Bastian and Rempala (2020); Di Lauro et al. (2022); Vossler et al. (2022)), we can interpret the system of Eq. (8] in terms of a statistical model for times to infection. To this end, as in KhudaBukhsh et al. (2020), we may consider $S_t:= x_S(t)$ as the survival probability of a typical node (i.e., the probability that a typical node who was susceptible at time $t = 0$ remains susceptible at time $t > 0$). Note that $S_0 = 1$ follows from the assumed initial conditions (9) and that $S_\infty > 0$, so $S_t$ is an improper survival function. In this section, we show how to derive a single autonomous differential equation for $S_t$ (or $x_S$) that allows numerical calculation of the survival probability for any $t\in [0, \infty )$ solely in terms of the network model parameters. We achieve this in several steps: First, we derive an integral that relates $x_{SS}$ and $x_{S}$. Because $\psi ''\left( x_{\theta }\right) / \psi '\left( x_{\theta }\right) ^2 = \kappa / x_S$ under the pairwise closure condition, we obtain:

27 $$\begin{aligned} \frac{\dot{x}_{SS}}{\dot{x}_S}= 2 \kappa \frac{x_{SS}}{x_S}. \end{aligned}$$

Integrating this and using the initial conditions $x_S(0) = 1$ and $x_{SS}(0) = \mu$ leads to:

28 $$\begin{aligned} x_{SS}(t) = \mu x_{S}(t)^{2 \kappa }. \end{aligned}$$

Second, the equations for $x_{S}$ can be rewritten as:

29 $$\begin{aligned} \dot{x}_{S} = - \beta \frac{x_{SI}}{x_{S}}x_{S} = -\beta x_D x_S, \end{aligned}$$

where $x_{D}=x_{SI}/x_{S}$ is considered a new variable for which an evolution equation is needed. Considering the derivative of $x_{D}$, and plugging in the expressions for $\dot{x}_{SI}$ and $\dot{x}_{S}$, we obtain:

30 $$\begin{aligned} \begin{aligned} \dot{x}_{D}&=\frac{\dot{x}_{SI}x_{s}-x_{SI}\dot{x}_{S}}{x_{S}^2} \\&= \frac{\beta \kappa x_{SS}x_{SI}-\beta \kappa x_{SI} x_{SI} - (\beta + \gamma ) x_{SI} x_{S} + \beta x_{SI}^2}{x_{S}^2} \\&= \beta \kappa \mu x_{s}^{2\kappa -1} \left( \frac{x_{SI}}{x_{S}}\right) - \beta \kappa \left( \frac{x_{SI}}{x_{S}}\right) ^2 - (\beta + \gamma ) \left( \frac{x_{SI}}{x_{S}}\right) + \beta \left( \frac{x_{SI}}{x_{S}}\right) ^2 \\&= \beta (1 - \kappa ) x_{D}^2 + \left( \beta \kappa \mu x_{S}^{2\kappa - 1} - (\beta + \gamma )\right) x_{D}. \end{aligned} \end{aligned}$$

Given that the equations for $x_{SI}$ and $x_{SS}$ no longer depend on $x_{\theta }$, the system now simplifies to three key equations:

31 $$\begin{aligned} \begin{aligned} \dot{x}_{S}&= -\beta x_{D}x_{S}, \\ \dot{x}_{I}&= \beta x_{D} x_{S} - \gamma x_{I}, \\ \dot{x}_{D}&= \beta (1 - \kappa ) x_{D}^2 + \left( \beta \kappa \mu x_{S}^{2\kappa - 1} - (\beta + \gamma )\right) x_{D}.\\ \end{aligned} \end{aligned}$$

Finally, we can manipulate these equations further. In particular, looking at

32 $$\begin{aligned} \frac{\dot{x}_{D}}{\dot{x}_{S}} + (1 - \kappa ) \frac{x_{D}}{x_{S}} = -\kappa \mu x_{S}^{2\kappa -2} + \frac{\beta + \gamma }{\beta } \frac{1}{x_{S}}, \end{aligned}$$

and considering $x_{D}$ as a function of $x_{S}$, we can use an integrating factor. This leads to:

33 $$\begin{aligned} -\dot{S}_t = {\left\{ \begin{array}{ll} \tilde{\beta }(1-S_{t}^{\kappa })S_{t}^{\kappa }+\frac{\tilde{\gamma }}{1-\kappa }S_{t} (1-S_{t}^{\kappa -1})+\tilde{\rho } S_{t}^{\kappa } &{}\text {if } \kappa \ne 1, \\ \tilde{\beta } (S_t - S_{t}^{2}) + \tilde{\gamma } S_{t} \log S_{t} + \tilde{\rho } S_{t} &{}\text {if } \kappa = 1, \end{array}\right. } \end{aligned}$$

where we now replaced $x_{S}(t)$ by $S_{t}$ and set $\tilde{\rho } = \beta \mu \rho$, $\tilde{\gamma }=\beta +\gamma$, and $\tilde{\beta }=\mu \beta$. As already noted, the initial condition inherited from (9) is $S_0 = 1$. Because necessarily $\dot{S}_\infty =0$, Eq. (33) implies that the limiting value $S_\infty >0$ has to satisfy

34 $$\begin{aligned} \tilde{\beta }(1-S_{\infty }^{\kappa })+\tilde{\rho } = \frac{\tilde{\gamma }}{1-\kappa }(1-S_{\infty }^{1-\kappa })&\qquad \text {if } \kappa \ne 1, \end{aligned}$$

35 $$\begin{aligned} \tilde{\beta } (1 - S_{\infty }) + \tilde{\rho } = -\, \tilde{\gamma }\, \log S_{\infty }&\qquad \text {if } \kappa = 1 . \end{aligned}$$

It is of interest to note that when the degree distribution is Poisson ($\kappa = 1$), then Eqs. (33) and (35) are identical to the ones known from the mass-action SIR dynamics.

This analysis shows that the dynamics of an SIR epidemic on a configuration model network with a PT degree distribution can be summarised with a single self-contained survival equation describing the evolution of survival probability $S_t$. This leads to the following interesting statistical consideration that was already noted for mass-action SIR models in KhudaBukhsh et al. (2020); Di Lauro et al. (2022); Vossler et al. (2022). Assuming that, over a time interval [0, *T*] where $T \le \infty$, we observe the times of infection $(t_1, \ldots , t_k)$ of a randomly selected set of *k* initially susceptible nodes of our network, we may write the approximate log-likelihood function as

36 $$\begin{aligned} \ell (\tilde{\beta },\tilde{\gamma },\tilde{\rho }\vert t_1,\ldots ,t_k) =\sum _{i=1}^k \log S_{t_i}-k\log (1-S_T). \end{aligned}$$

To obtain quantities other than $S_t$, evaluation of additional ODEs is needed as discussed, for instance, in KhudaBukhsh et al. (2020) or Khuda Bukhsh et al. (2022). Let us also note that, since the DSA and Volz models are equivalent (see Lemma 1 in the Appendix), the representation $S_t$ in  (33) can be similarly derived directly from Volz’s model (6).

## Discussion

Over the last two decades, two types of disease network models have emerged as particularly relevant in many practical applications (including the recent COVID-19 pandemic, see KhudaBukhsh et al. (2023)): the so-called pairwise Rand (1999b); Keeling (1999) and edge-based Volz (2008); Miller et al. (2012) approaches. More recently, a version of an edge-based approach, dubbed DSA, was proposed in Jacobsen et al. (2018); KhudaBukhsh et al. (2020) to facilitate statistical inference.

In this paper, we have shown that the three approaches are equivalent and asymptotically exact under the assumption that the contact network underlying the spread of disease is a configuration model random graph with one of the three Poisson-type (PT) degree distributions: Poisson, binomial, or negative binomial. Perhaps more interestingly, we have shown that the pairwise closure for an epidemic on a configuration model network is exact if and only if the ratio of mean excess degree to mean degree for susceptible nodes remains constant over time (as the susceptible nodes are depleted). This condition holds if and only if the degree distribution is PT. As an interesting corollary of our results, we obtained a single equation representation of the pairwise model that allows parameter estimation from time series data marginalised over the network degree distribution. This finding is practically useful as it allows, for instance, statistical inference based solely on the disease incidence data as in the classical, homogeneous SIR models. Because these statistical methods are based on survival times in susceptible individuals, statistical inference can be based on observation of a random sample of the population, which could allow more accurate tracking of future epidemics.

## Data Availability

The datasets generated and/or analysed during the current study are available upon reasonable request from the corresponding author.

## A Summary of notation

The following notation is used throughout the paper and in particular in the next section.

- $\beta :=$ the force of infection per infectious neighbour (i.e., the constant rate at which infectious nodes infect a neighbour).
- $\gamma :=$ the recovery rate (i.e., the constant rate at which infected nodes become recovered).
- $p_{k}:=$ the probability that a node will have degree *k*.
- $\psi (x):=$ the probability generating function for the degree distribution $\left\{ p_{k}\right\}$.
- $x_S:=$ the proportion of nodes susceptible at time *t*.
- $x_I:=$ the proportion of nodes infectious at time *t*
- $x_{AB}:=$ the proportion of *AB*-type edges at time *t*
- $\mathcal {A}_{X}:=$ the set of arcs (*i*, *j*) such that node *i* is in set *X*.
- $M_{X}:=$ the proportion of arcs in set $\mathcal {A}_{X}$.
- $\mathcal {A}_{X Y}:=$ the set of arcs (*i*, *j*) such that $i \in X$ and $j \in Y$.
- $M_{X Y}:=$ the proportion of arcs in set $\mathcal {A}_{XY}$.
- $\theta :=$ the probability that infection has not crossed a randomly chosen edge at time *t*, also denoted by $x_{\theta }$ in some of the models.
- $p_{I}:= M_{SI} /M_{S}$, the probability that an arc (*i*, *j*) with a susceptible *i* has an infectious *j*.
- $p_{S}:= M_{SS} /M_{S}$, the probability that an arc (*i*, *j*) with a susceptible *i* has a susceptible *j*.

## B Equivalence of Volz’s and DSA models

### Lemma 1

The Volz model (6)–(7) and the DSA model (8)–(9) are equivalent.

### Proof

While our starting point is the system of Volz’s original set of Eq. (6), it is useful to recast these over a different state space in order to show equivalence with the DSA system in Eq. (8). Here, we move from the state space in terms of ($\theta , p_{I}, p_{S}$) to a state space in terms of ($\theta =x_{\theta }, x_{SI}, x_{SS}$), where $x_{SI}$ and $x_{SS}$ are simply the limiting counts of all *SI* and *SS* -type edges (counted in both directions) scaled by *N* (the number of nodes in the network) with the limit taken as $N \rightarrow \infty$.

As alluded to above, $\theta$ and $x_{\theta }$ have exactly the same interpretation, but we denote them differently to differentiate consistently between models over different state spaces. We now proceed to show how one moves from the original Volz model to the DSA equations. We start by showing that the first equation in (6) is equivalent to the first equation in (8). Starting from (6), and taking into account that $M_{SI}=x_{SI} / \psi '(1)$ and that $M_{S} = x_\theta \psi '(x_\theta ) / \psi '(1)$ as shown in Volz (2008), we obtain

37 $$\begin{aligned} \dot{\theta } = -\beta p_{I}\theta = -\beta \frac{M_{SI}}{M_S} \theta = -\beta \frac{\frac{x_{SI}}{\psi '(1)}}{\frac{x_{\theta } \psi '(x_{\theta })}{\psi '(1)}} x_{\theta } = -\beta \frac{x_{SI}}{\psi '(x_{\theta })} = \dot{x}_\theta . \end{aligned}$$

Showing the equivalence between the other equations requires an extra step. That is, the evolution equations for $p_I$ and $p_S$ need to be rewritten explicitly in terms of *SI*- and *SS*-type edges. In Volz (2008), it was shown that the equations for $M_{SI}$ can be written as

38 $$\begin{aligned} \dot{M}_{SI} = \beta p_{I} \left( p_{S} - p_{I}\right) \theta ^{2} \frac{\psi ''(\theta )}{\psi '(1)} - (\beta + \gamma ) M_{SI}. \end{aligned}$$

Using the expressions for $M_{SI}$ and $M_S$ in terms of the DSA model parameters above and the fact that $M_{SS} = x_ss / \psi '(1)$, we get $p_I = x_{SI} / (x_\theta \psi '(\theta ))$ and $p_S = x_{SS} / (x_\theta \psi '(x_\theta ))$. Substituting these into (38), we get

39 $$\begin{aligned} \begin{aligned} \dot{M}_{SI}&= \beta p_{I} \left( p_{S} - p_{I}\right) \theta ^{2} \frac{\psi ''(\theta )}{ \psi '(1)} - (\beta + \gamma ) M_{SI} \\ {}&= \beta \frac{x_{SI}}{x_\theta \psi '(x_\theta )} \left( \frac{x_{SS}}{x_\theta \psi '(x_\theta )} - \frac{x_{SI}}{x_\theta \psi '(x_\theta )}\right) \frac{x_\theta ^2 \psi ''(x_\theta )}{\psi '(1)} - (\beta + \gamma ) \frac{x_{SI}}{\psi '(1)} \\&= \frac{x_{SI}}{\psi '(1)} \left[ \beta (x_{SS} - x_{SI}) \frac{\psi ''(x_\theta )}{\psi '(x_\theta )^2} - (\beta + \gamma )\right] \\&= \frac{\dot{x}_{SI}}{\psi '(1)}. \end{aligned} \end{aligned}$$

Thus, Eq. (6) is equivalent to the equation for $x_{SI}$ in (8). Following Volz (2008), the evolution equation for *SS*-type edges can be rewritten to

40 $$\begin{aligned} \dot{M}_{SS} = -2 \beta p_I p_S \theta ^2 \frac{\psi ''(\theta )}{\psi '(1)}. \end{aligned}$$

Making the same substitutions as before, we get

41 $$\begin{aligned} \begin{aligned} \dot{M}_{SS}&= -2 \beta \frac{x_{SI}}{x_\theta \psi '(x_\theta )} \frac{x_{SS}}{x_\theta \psi '(x_\theta )} \frac{x_\theta ^2 \psi ''(x_\theta )}{\psi ''(1)} \\&= -2 \beta x_{SI} x_{SS} \frac{\psi ''(x_\theta )}{\psi '(x_\theta )^2 \psi '(1)} \\&= \frac{\dot{x}_{SS}}{\psi '(1)}, \end{aligned} \end{aligned}$$

which shows that (40) and the equation for $x_{SS}$ in (8) are equivalent. Since the remaining equations in both systems rely on the first three equations that we have just shown to be equivalent, the Volz and DSA models are equivalent under their respective initial conditions. $\square$

## Footnotes

1 The construction technically permits the inclusion of self-loops and unmatched edges, but in practice, these cases are infrequent and can be disregarded as $N \rightarrow \infty$, provided that the degree distribution has a finite variance. This has been discussed in detail in Section 7 of Van Der Hofstad (2016).
